# Development of in situ dosimetry for FLASH proton radiotherapy via organic scintillating fibers

**DOI:** 10.1093/bjr/tqag152

**Published:** 2026-06-19

**Authors:** Codey Olson, Jacob Strong, Adam Paxton, Geoff Nelson, Edward Cazalas

**Affiliations:** Department of Civil and Environmental Engineering, University of Utah Nuclear Engineering Program, Salt Lake City, UT 84112, United States; Department of Civil and Environmental Engineering, University of Utah Nuclear Engineering Program, Salt Lake City, UT 84112, United States; Department of Radiation Oncology, Huntsman Cancer Institute, Salt Lake City, UT 84112, United States; Department of Radiation Oncology, Huntsman Cancer Institute, Salt Lake City, UT 84112, United States; Department of Civil and Environmental Engineering, University of Utah Nuclear Engineering Program, Salt Lake City, UT 84112, United States

**Keywords:** FLASH, dosimetry, GEANT4, proton, radiotherapy

## Abstract

**Objectives:**

This work presents the design, development, and initial proof-of-concept testing of a dosimetry device intended for FLASH radiotherapy.

**Methods:**

The system incorporates organic scintillating fibers coupled to a silicon photomultiplier (SiPM) and an FPGA-based readout, with fiber geometry optimized to minimize perturbations to a clinical proton beam, as confirmed through measurements at the Huntsman Cancer Institute (HCI).

**Results:**

Tests using a portable X-ray unit demonstrated a strong correlation between integrated charge and delivered dose (up to ∼35 mrem s^−1^) and showed that the detector can respond on nanosecond timescales, enabling rapid safety interlock triggering with minimal latency.

**Conclusion:**

Preliminary proton irradiations at HCI further indicated that the device can resolve individual beam pulses, a capability valuable for both FLASH research and conventional radiotherapy.

**Advances in knowledge:**

These results highlight the need for continued refinement of the experimental setup, readout electronics, and detector performance to fully realize real-time, in situ dose and pulse monitoring.

## Objectives

There is ongoing research in radiation therapy advancing toward ultrahigh-dose rates over very short time scales. In particular, advancements toward clinical applications and research using FLASH RT^1–4^ are increasingly coveted as potential benefits are realized.^5–7^ FLASH RT uses very high dose rates, usually from 10 to 100 Gy s^−1^. Such dose rates are possible as they are delivered as micro-pulses over very short timescales (typically on the millisecond-to-nanosecond scale), but these pulses are dispersed in time to give typical FLASH RT dose rates for the whole procedure.^8–12^ Numerous studies operating in this regime have shown a decrease in healthy cell complications while maintaining the tumor control probability (TCP) in comparison to conventional RT treatments.^13–17^ Thus, the adoption of this dosimetry method may enhance the efficacy of RT into the future.

FLASH RT has been recently promising in progress and understanding, yet there remain challenges. For example, how is this treatment method to be monitored for required dose accuracy and beam timing to ensure patient safety?^18^^,^^19^ The dose rates expected during delivery limit the response time of monitoring equipment, producing a reduction in the acceptable margin of error in treatment machines.^20^^,^^21^ Conventionally, ionization chambers are used for online dose delivery monitoring and perform sufficiently for dose rates in clinical RT treatments. However, during a FLASH RT treatment, instantaneous dose rates on the order of 10^5^-10^7^ Gy s^−1^ readily saturate ionization chambers and induce recombination effects.^22^ Work has been undertaken to anticipate these effects,^23^ but the predictive or empirical models utilized so far are not to clinical standards. We also refer to the terms “conformal FLASH optimization” and “peer conformal FLASH dose rate.” Here, conformal FLASH optimization is about tailoring proton beam delivery to achieve FLASH dose rates while maintaining tumor conformality. The concept of peer conformal FLASH dose rate provides standardized definitions (average, instantaneous, and conformality-weighted) to ensure reproducibility and comparability across studies.^24^ Our goal is to use peer conformal FLASH dose rates.

Numerous works have investigated updated systems and equipment to accurately monitor dose delivery in real time, which could have applications in FLASH treatments.^25–43^ However, the general response times are much too slow to allow for online monitoring and intervention.^30^^,^^32^^,^^33^ Some can perform adequate temporal resolution but these would significantly alter the primary beam characteristics during measurement.^26^^,^^28^^,^^29^^,^^32^^,^^34^^,^^37^^,^^39^ Thus, these measurement systems are not adequate for in situ beam monitoring. The current work creates and studies a measurement system for integration into an RT machine for online dose monitoring and intervention timescales at FLASH rates.

In this report, we investigate a custom scintillating fiber-based dosimeter for in situ FLASH RT dosimetry. Our design is intended for use in proton FLASH RT but may be implemented for other RT modalities and radiation types. Here, we outline proof-of-concept design and focus on the temporal response of the system. Our project goal is for testing with a clinical machine with FLASH capabilities. With RT machine manufacturer support, we plan to gain access to internal components, especially those of a Mevion proton therapy system at HCI (Huntsman Cancer Institute) at the University of Utah. In our study, we utilize 2 radiation fields–a desktop X-ray machine and the synchrocyclotron at HCI—each machine emphasized testing of different characteristics of our system. Our study reports on the preliminary design of the dosimeter and provides information to improve on our system with regard to FLASH RT in situ monitoring.

## Methods

### Radiation sources

We utilized a desktop X-ray machine (TUB00154-SA-M06) produced by Moxtek Inc., 12 W power production, with selectable beam current and potential (1-300 μA and 10-70 kVp, respectively). A molybdenum target is used and the machine is controlled via USB connection to a laptop with control software provided by the manufacturer. Due to the machine’s electronic programming and computer control, however, the on/off times are limited to a resolution of 1 s, and therefore it is recognized that this X-ray source does not adequately represent the beam time structure of a clinical machine.^44^

The machine output was constant at 60 kVp during all irradiations, with a varying beam current to simulate a changing dose rate to the scintillating fiber. The machine includes a digital interlock, which was used during testing protection of the irradiation area and lab. The interlock was modified from its original form to allow for the integration of a switch and programmable dose threshold, which is discussed further in “Hardware Description” and “Firmware.” The integration of this switch enables the study of the temporal response of the system, which was used to estimate its feasibility as a monitoring device for FLASH treatments. The X-ray machine is shown in Figure SM1.

Our study also used a Mevion S250i proton therapy system located at the HCI. The system, used for clinical cancer treatments, utilizes a synchrocyclotron to produce a narrow beam of protons at an energy of 230 MeV. Protons energy and emission are controlled with a set of 18 polycarbonate range selector plates of 6 different thicknesses. An appropriate selection of plates gives the range of protons desired and is controlled with a depth resolution of ∼ 2 mm within the patient. A radio frequency (RF) controller is utilized for beam structuring and maintains a frequency of 750 Hz frequency with pulses anywhere from 1 to 20 μs in length and total charges in the range of 1 to ∼15 pC, respectively. The beam typically delivers 8-pC pulses for the majority of treatment with pulses being broken into a set of “pulselets.” On average, these pulselets include a series of 4 pulses of lower charge that are used to slowly approach the prescribed dose and avoid over or under delivery. The relation between the pulse length and charge per pulse is well understood for the machine; yet, there are uncertainties of total charge delivered per pulse as a function of the pulse width. Other studies have also completed pulse structure verification.^45^ This concern is outside the scope of this paper, and we acknowledged that a study on how variation in charge per pulse affects practical calibrations is deserved. The testing facility is the treatment vault of the Mevion system at HCI.^46–48^ This is shown in Figure SM2.

The HCI Mevion proton beam machine is used for clinical treatments at UofU. Thus, manipulation of beam characteristics to produce FLASH rates was not possible at the time of this work. Instead, the machine was utilized in clinical mode with the objective of demonstrating our dosimeter’s ability to monitor the beam dose.

### Simulation efforts

Our dosimetry system aims at in situ monitoring of dose during FLASH treatments. We performed preliminary simulation studies using GEANT4 simulations^49^ to estimate the energy absorption and divergence of the primary beam with a scintillator in-beam. GEANT4 V 10.07 version was used for simulations performed on the Center for High Performance Computing modules at the University of Utah (CHPC), using a total of 64 cores with hyperthreading enabled, for a total of 128 simulated threads. The QGSP_BIC_AllHP physics list was used, which included all necessary hadronic interactions with high precision enabled. A total of 10^7^ particles were simulated for each geometry and energy—no additional bias techniques were used.

The Mevion system beam was recreated in our simulations with a 230 MeV primary proton beam, 18 range selector plates, an adaptable nozzle location between 3.6 and 33.6 cm from isocenter, and a nickel adaptive aperture. The scintillator fibers in the simulations formed a 5 cm × 5 cm plane (of fiber thickness), placed adjacent to the beam exit nozzle (orthogonal to the beam propagation), and a beam stop of tissue was placed downstream with its front face located at room isocenter. This allowed for the Bragg peak to be simulated in the GEANT4 geometry, which could be compared to that of measured and simulated results performed during beam commissioning of the system at the HCI. A detailed description of the geometry and source code can be made available upon request.

### Hardware setup

For simulations and experimental work, we use a 0.5 mm diameter fiber of BCF-12 from Luxium Solutions.^50^ We chose this scintillator based on its short decay time (3.3 ns) and long attenuation length (210 cm) that allow for fast reporting times and low light loss during transmission. The fibers were cut to ∼10 cm lengths. One end of each of these fibers was terminated in an SMA connector and coupled using optical grade epoxy (both components from Industrial Fiberoptics^51^) to the ends of the SMA connectors. The fiber ends were cleaned and polished with a polishing kit from Industrial Fiberoptics to minimize distortion of light transmission at junctures. Attached to these were ∼150 cm (approximately 5 ft) long and 0.5 mm in diameter plastic optical fiber (POF) cables (with sheath) for signal transmission. Both terminating ends of the POF cables utilized similar SMA connectors and epoxy to the terminating end of the scintillating fiber, and these POF cable connection points were similarly cleaned and polished. The terminated ends of the scintillating fibers were then mated with the POF cables for signal transmission. This cable mating was accomplished using an active device coupler was used at the junction. This connection device offered reliable and reproducible mating of the 2 fibers along with use of polishing and optical grease. The conventional POF and scintillating fibers are shown in Figure 1 with the active device coupler.

**Figure 1:**
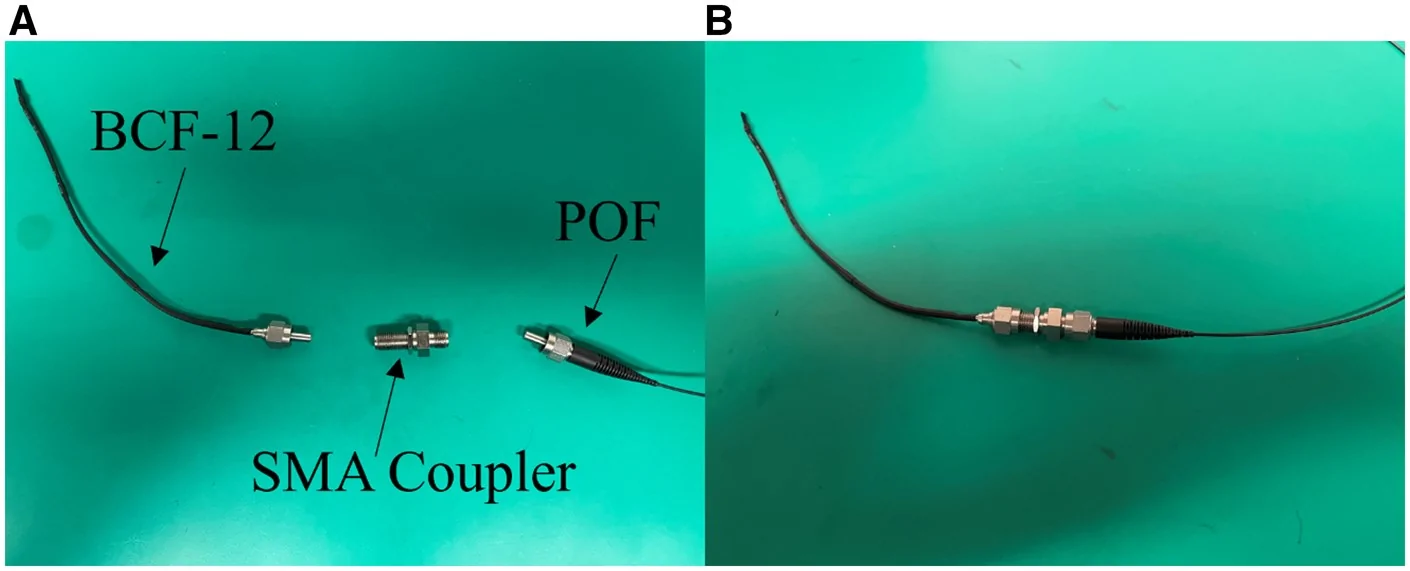
(A) The 0.5 mm BCF-12 scintillating fiber optic cable and ESKA POF cable and SMA coupler. (B) This shows our coupling between the scintillating fiber and POF.

A study comparing the attenuation of the fiberoptic cables and their junctions was performed prior to irradiations to understand any variations in quality of polishing and preparation of each junction. We utilized a simple blue LED for this study, which was attached to an SMA connector using optical grease and electrical tape in order to allow for direct mating to all POF cables. (We utilized a blue LEB to simulate the light emissions from the scintillating fiber [peak emission wavelength of 435 nm.^50^]) Each of these POF cables, once mated to the blue LED, were then connected to a photodiode, which was itself attached to an SMA connector, using the SMA coupler shown in Figure 1. Together, this arrangement of a blue LED, 2 SMA connection junctions, and a photodiode was used to compare the photo resistance values measured for each of the prepared POF cables, and this comparison was used as an estimation of variability in the attenuation of each of the cables. It was found that the variation for photo resistance values for all cables varied by less than 10%. For the purposes of this study, this 10% margin of error was deemed adequate, especially considering a single fiber was tested in the proton beam, not requiring all of the POF cables to be identical.

A silicon photomultiplier (SiPM) was used to measure the fiber-optic signals from the fibers. We used a model C13369-3050EA-04 multi-pixel photon counter (MPPC) from Hamamatsu. This device has 16 analog MMCX connection output channels corresponding to the 16 channels of the SiPM array.^52^ We 3D-printed, in-house, an SMA-compatible adaptor to attach to the MPPC array channel, allowing for fibers to be properly and reproducibly moved into place. Optical grease was used at junction points to optimize light transfer. Figure 2 displays the MPPC and SMA adapter.

**Figure 2:**
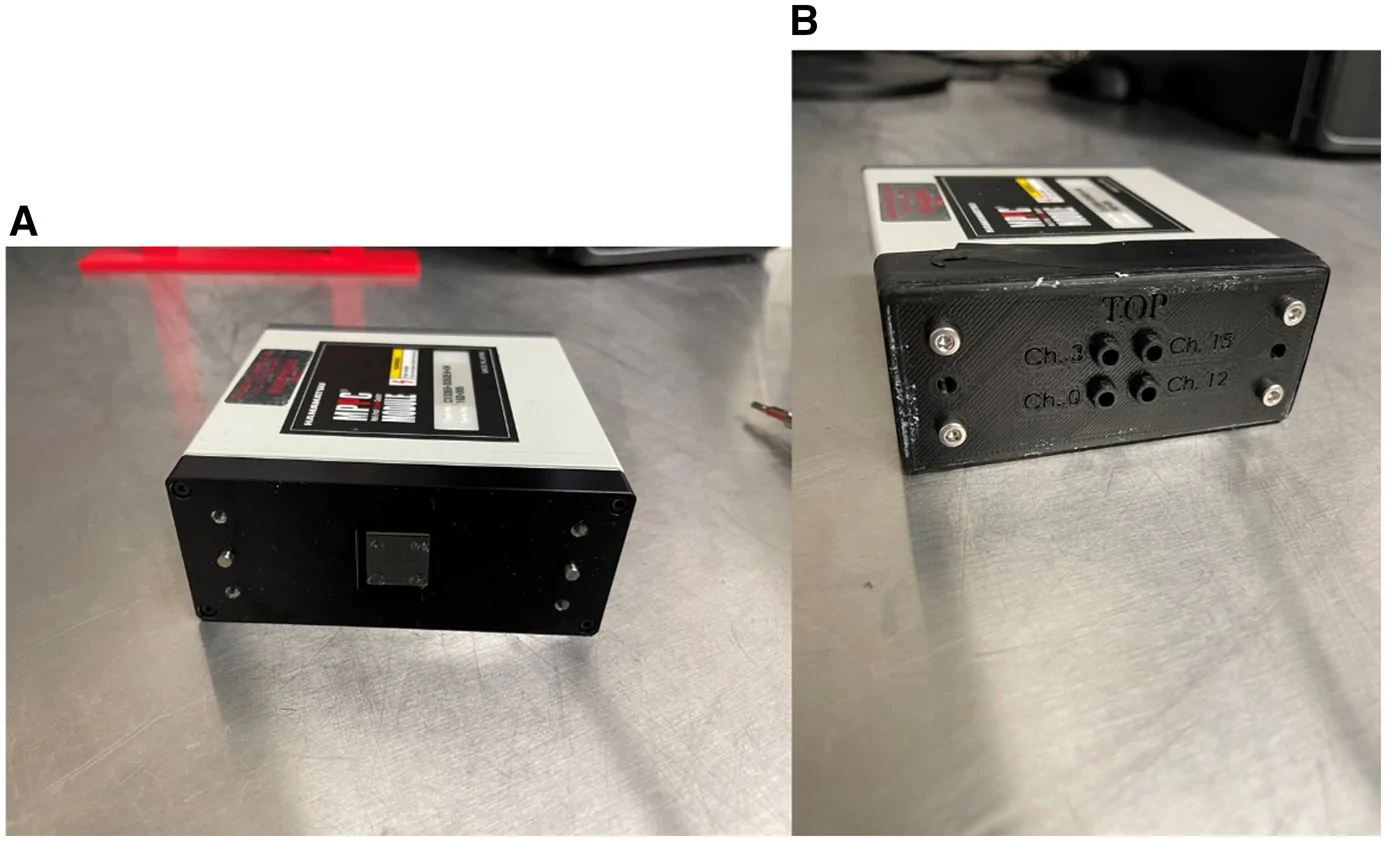
(A) The MPPC array developed by Hamamatsu showing the SiPM (without mate cover), and (B) our 3D printed SMA adaptor connect the POF to the face of the MPPC array. Channel numbers are shown with each channel signal individually provided by the MPPC.

The MPPC signal is fed into a Field Programmable Gate Array (FPGA). We used a CAEN model DT5550 32 Channel Programmable Readout System with custom firmware.^53^ The DT5550 board employs a 68-pin VHDCI connector for analog signal input from the MPPC array. This required us to utilize a 68-pin breakout board for signal transmission into the FPGA. Both single-ended and differential signals are possible, however we used single-ended signals for this work. Figure 3 displays the DT5550 FPGA and breakout board.

**Figure 3:**
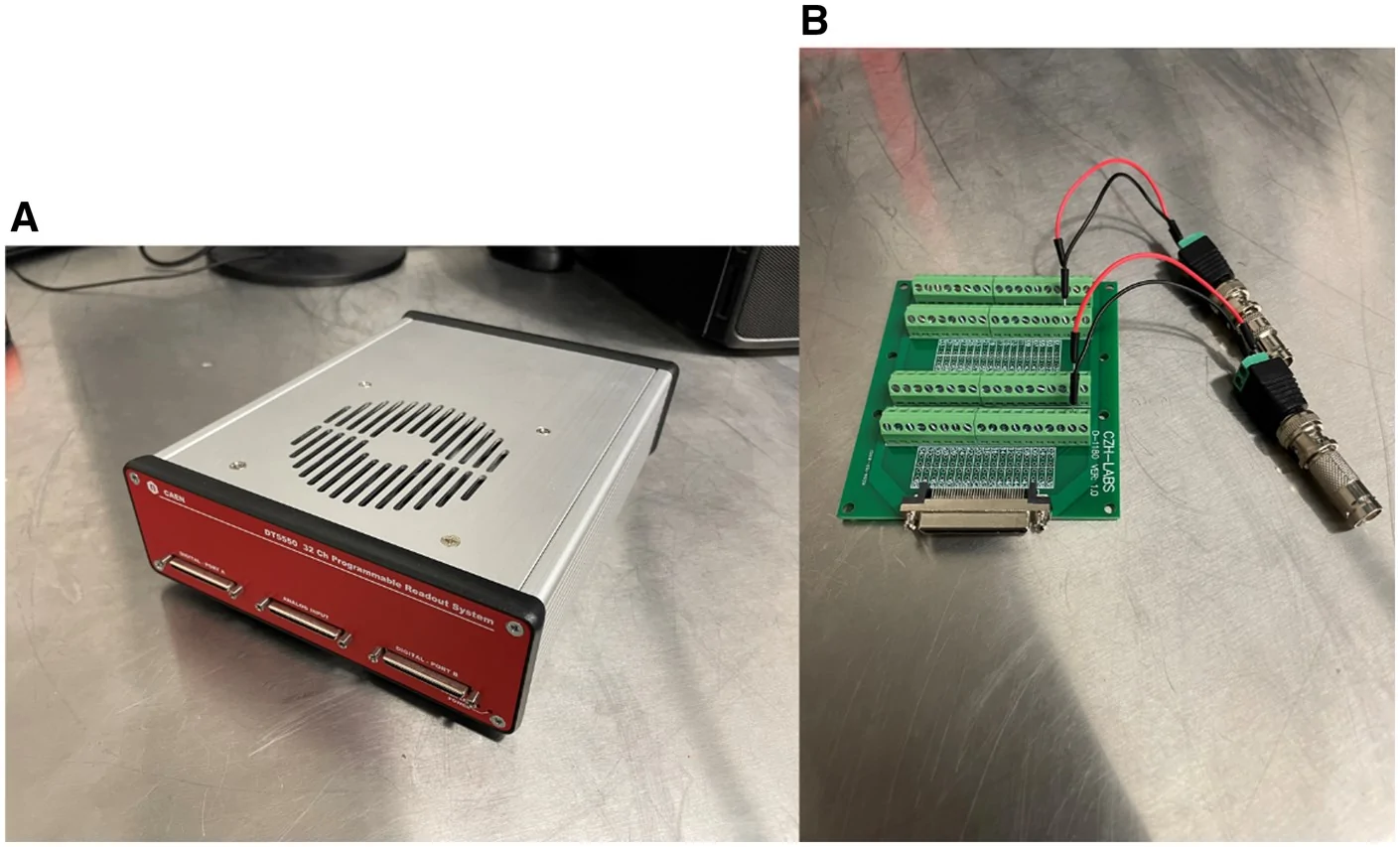
(A) DT5550 FPGA (CAEN), and (B) 32 channel breakout board (converts to VHDCI cable).

The interlock on our X-ray machine was employed to test the timing resolution of the FPGA system. The X-ray machine interlock requires a 5 V digital signal, which stops X-ray production. For this test, a digital 5 V signal from the FPGA given when a preset integrated dose limit. This signal was sent from a general-purpose input/output (IO) on the FPGA to the X-ray interlock circuit. We note that there was a mismatch in the logic voltages of the 2 machines. The FPGA logic output signal was at 2.1 V but 5 V was required. Thus, a logic level shifter was implemented, allowing for common logic between the 2 devices. For this, we used a 5 V and 2.1 V power supply, along with 10 kΩ pull-up resistors and a BS170 transistor. We provide the full schematic of this setup in Figure SM3.

### Dosimetry

We used an Instadose+ from Mirion for dosimetry during X-ray testing of the device, and the dosimeter was placed at the same location as the scintillating fiber. We averaged 3 10-second irradiations to find the average dose rates delivered from the X-ray machine at 4 current levels, from which, we measured linearity of the system per dose rate incident upon the device. We placed the dosimeter in line with the X-ray beam at a distance of ∼ 1 mm. The setup of this dose calibration is shown in Figure 4.

**Figure 4:**
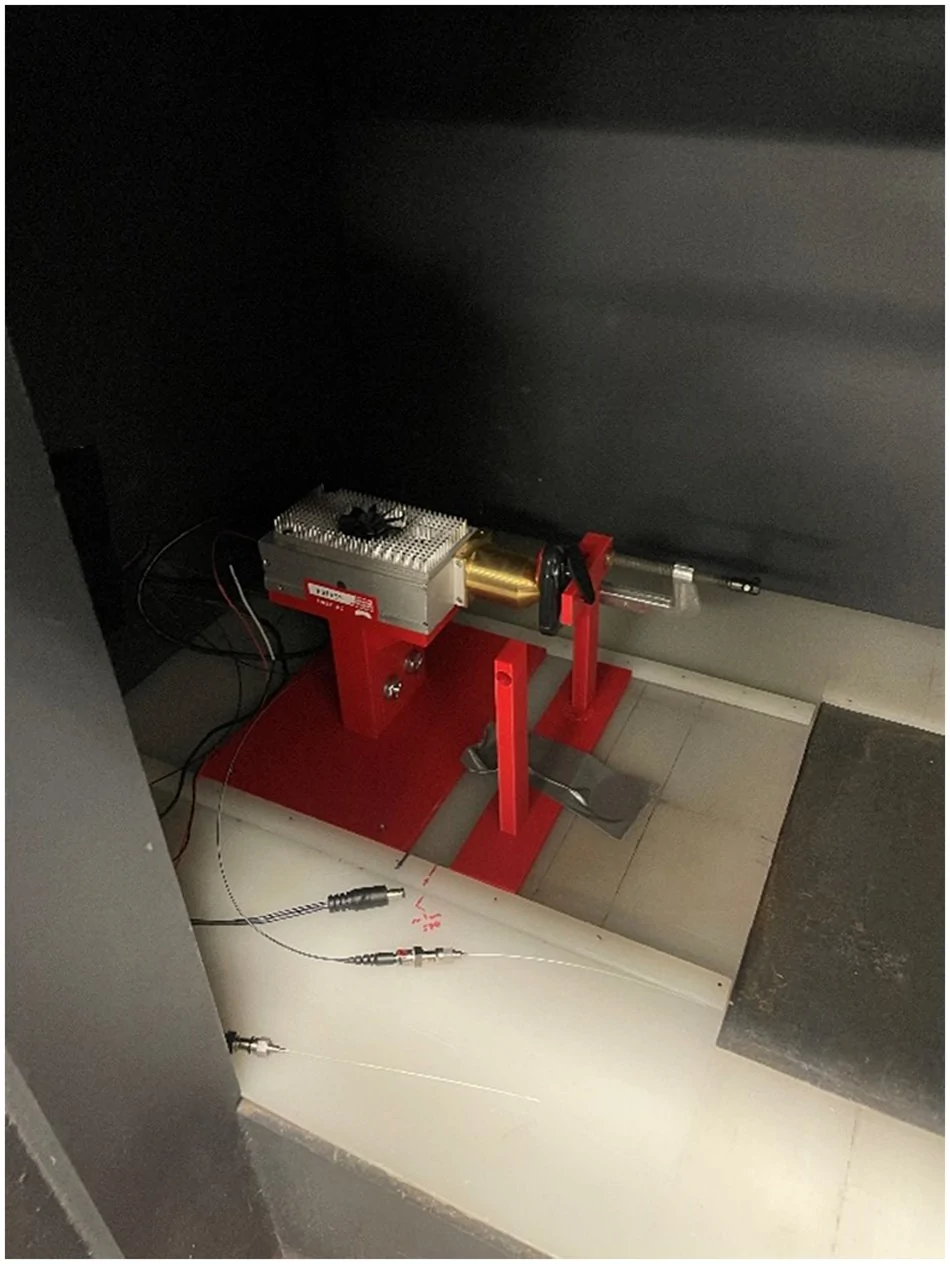
Setup of X-ray machine used for dosimetry. We place the full setup inside a large steel light-tight radiation shielding cave for radiation protection.

The dose in the preliminary experiment with the proton beam was dependent on the length of the scintillating fiber. With longer lengths giving higher light yields. This particular experiment was a proof-of-concept measurement. In future efforts, the length of the fiber will be correlated with beam exit full field view. While there are metal components that may undergo transmutation, they are outside of the beam directly and far enough away to minimize neutron absorption. Likelihood of creating radioactivity is minimal compared to the rest of the room environment.

### Irradiations

We performed X-ray machine and Mevion system (proton) irradiations on our system. In these tests, we placed a scintillating fiber perpendicular to the incoming beam with a POF cable delivering the light to an MPPC array outside of the radiation beam. For X-ray experiments, both X-ray machine and fiber were placed in a steel radiation shielding enclosure with a POF cable accessed via a port in the shield.

The X-ray machine experiments used 10-s irradiations, with 5 s between irradiations. Different amounts of beam current were tested to represent different dose rates. For the proton beam irradiation at the UofU HCI, we employed different proton beam energies. In total there were 4 different energies with 3 trails for each energy. Additionally, each beam energy was measured at 2 different prescribed doses of 10 MU and 100 MU, corresponding to ∼156 pC and ∼1560 pC, respectively. The proton beam was pencil geometry and our fiber was placed in the beam with the beam nozzle fully extended. Thus, there was a distance of 3.6 cm between our measurement fiber and the exit window of the nozzle. We display our setup in Figure 5.

**Figure 5:**
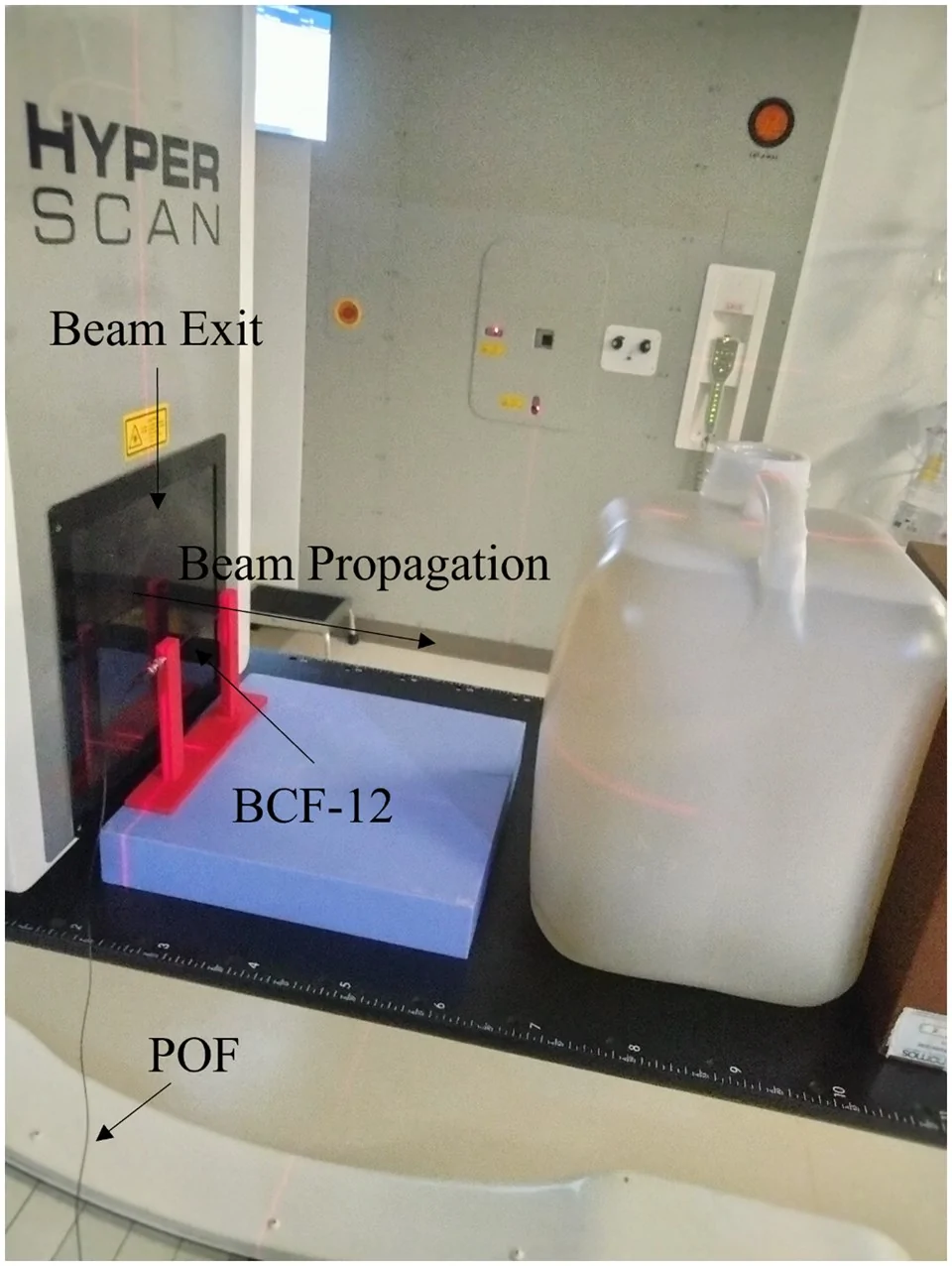
Setup of our detector at UofU HCI proton beam facility.

## Results

### Simulated beam perturbations

HCI beam characteristics were determined by comparing of simulated and experimental measurements. Bragg peak location was of interest, as it is indicative of a need for correction in prescribed energy during treatments. We performed GEANT4 simulations and compared these to measured values of 4 selected energies, shown in Figures 6 and 7. “G4” values show the GEANT4 results with a 1 mm scintillator and “Meas,” “MonteCarlo,” and “PencilBeam” represent the beam from commissioning at HCI with no fiber. It should be noted that these data were all provided by HCI, and the measurements/simulations shown, were not performed for this work but rather were provided as a benchmark for the current GEANT4 simulation. A 1 mm scintillator was found to modify the distal (90%) range of the Bragg peak by less than 1% compared with the average of the measured and calculated values provided by HCI for their proton machine. This would increase the dose seen prior to the Bragg peak and is apparent at lower energies (eg, 44.61 MeV in Figure 6). Thus, using a 1-mm scintillator, a 2-layer array of fibers to form a gridded layout for spatial information of the beam will not alter proton beam energy or angulation in any fractional way that will alter beam dosimetry or delivery location. In addition, the fibers can be incorporated into and accounted for in dosimetry planning simulations.

**Figure 6:**
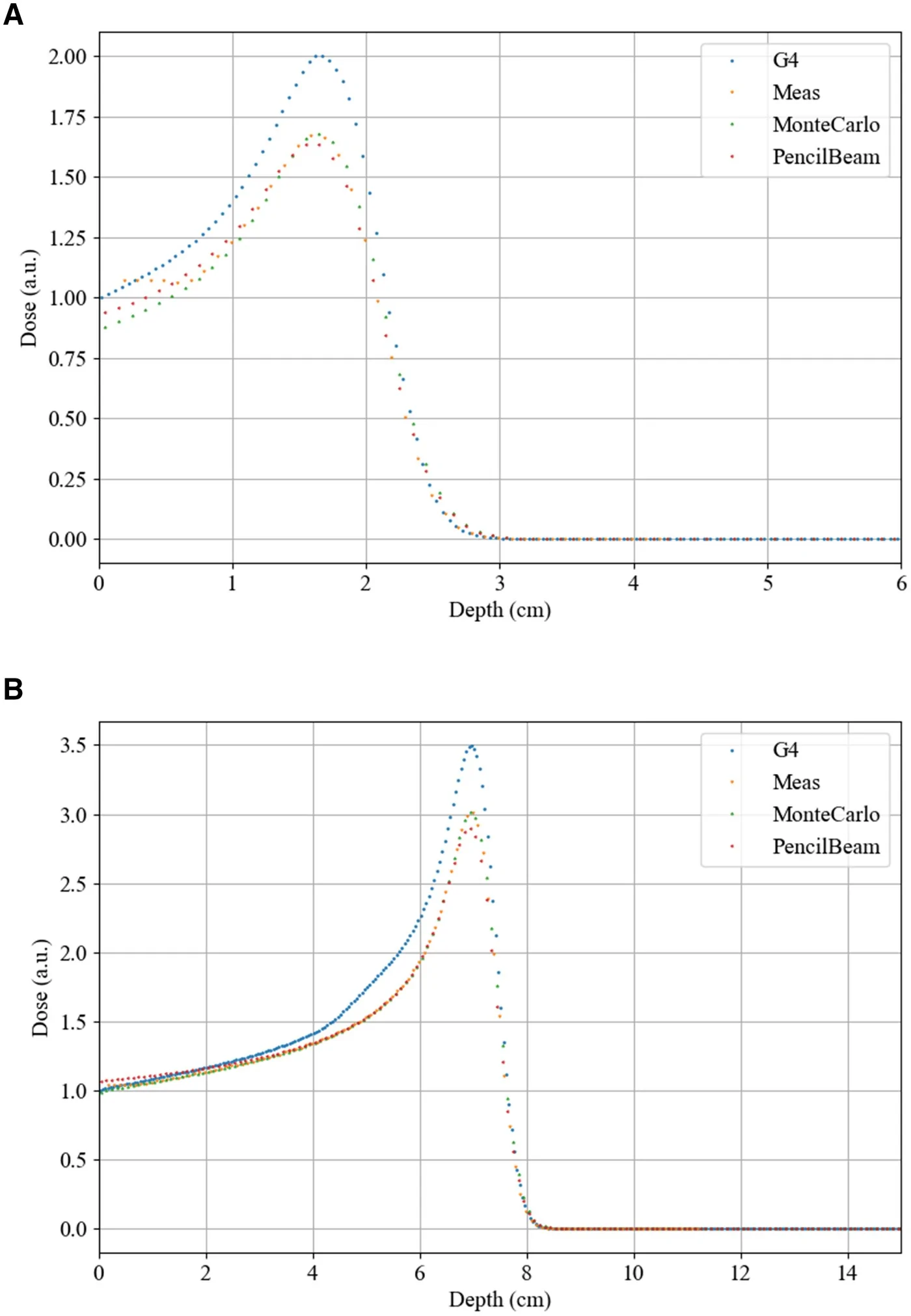
The Bragg peaks for protons of (A) 44.61 MeV, and (B) 96.64 MeV. “G4” denotes the GEANT4 simulation results compared to “Meas,” “PencilBeam,” and “MonteCarlo” which represent measured (“Meas”) and computed (“PencilBeam,” “MonteCarlo”) results with no scintillator. Data for “Meas,” “MonteCarlo,” and “PencilBeam” are provided by UofU HCI Proton Beam Facility.

**Figure 7:**
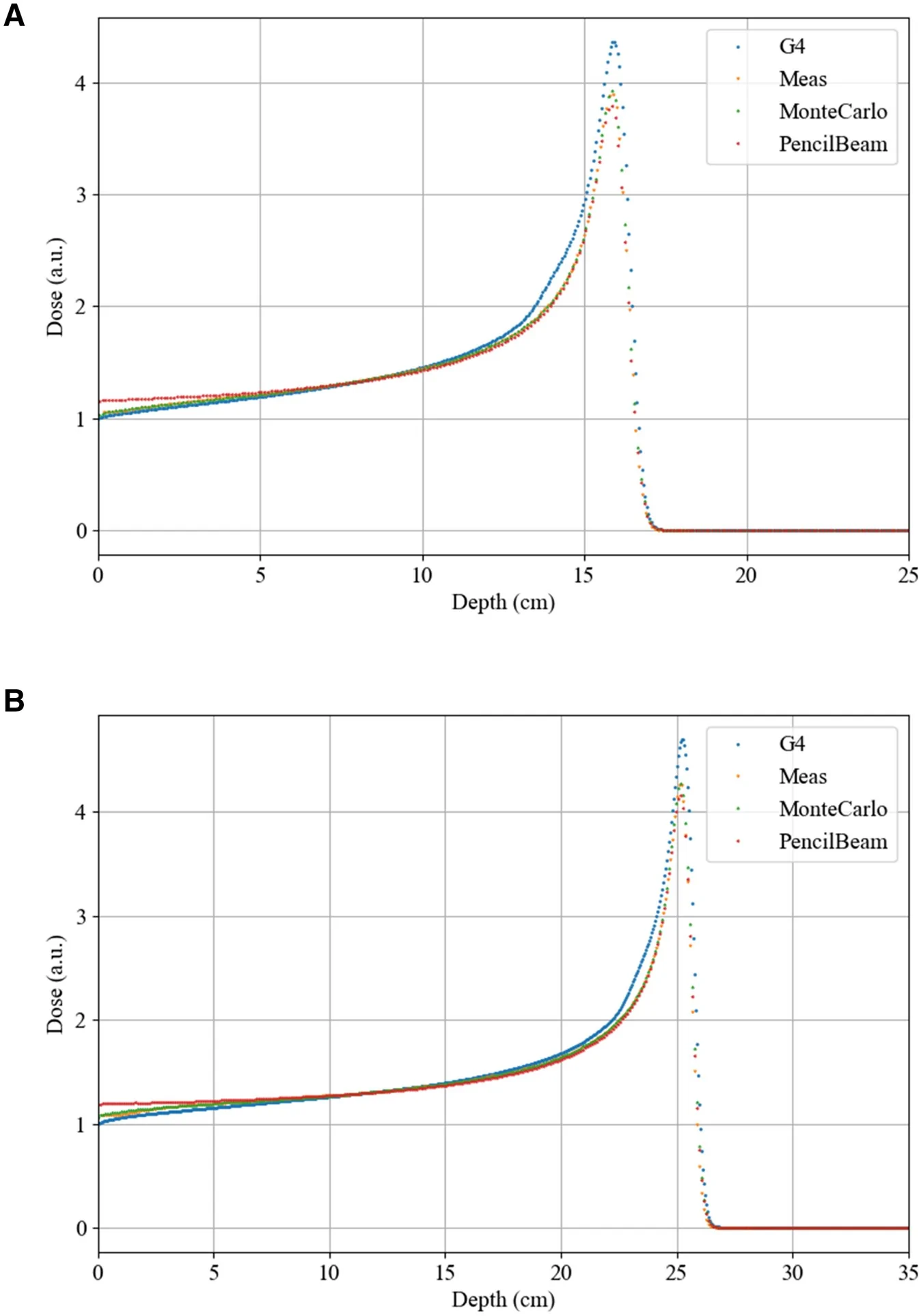
The Bragg peaks for protons of (A) 152.87 MeV and (B) 198.26 MeV. “G4” represents the GEANT4 simulationm results compared to “Meas,” “PencilBeam,” and “MonteCarlo” which represent measured (“Meas”) and computed (“PencilBeam,” “MonteCarlo”) results with no scintillator. Data for “Meas,” “MonteCarlo,” and “PencilBeam” are provided by UofU HCI Proton Beam Facility.

The Bragg peak parameters are important, however another key characteristic of the proton beam is the spot profile. Figures 8 and 9 show the X-Y (2d) cross sections of the proton beam at the same energies exhibited in Figures 6 and 7. We observe an very small increase in the penumbra of the spot profile. However, these are nearly identical to those in measured and simulated data, indicating minimal effects of the measurement fibers on the spatial profile of the beam. We acknowledge here that other detectors have worked for proton RT measurements, including dose validation.^24^ While reported detectors state time resolution, here, for safety implementation, full time of signal processing (from input to output) is most important in order to send a “stop” signal to the RT machine.

**Figure 8:**
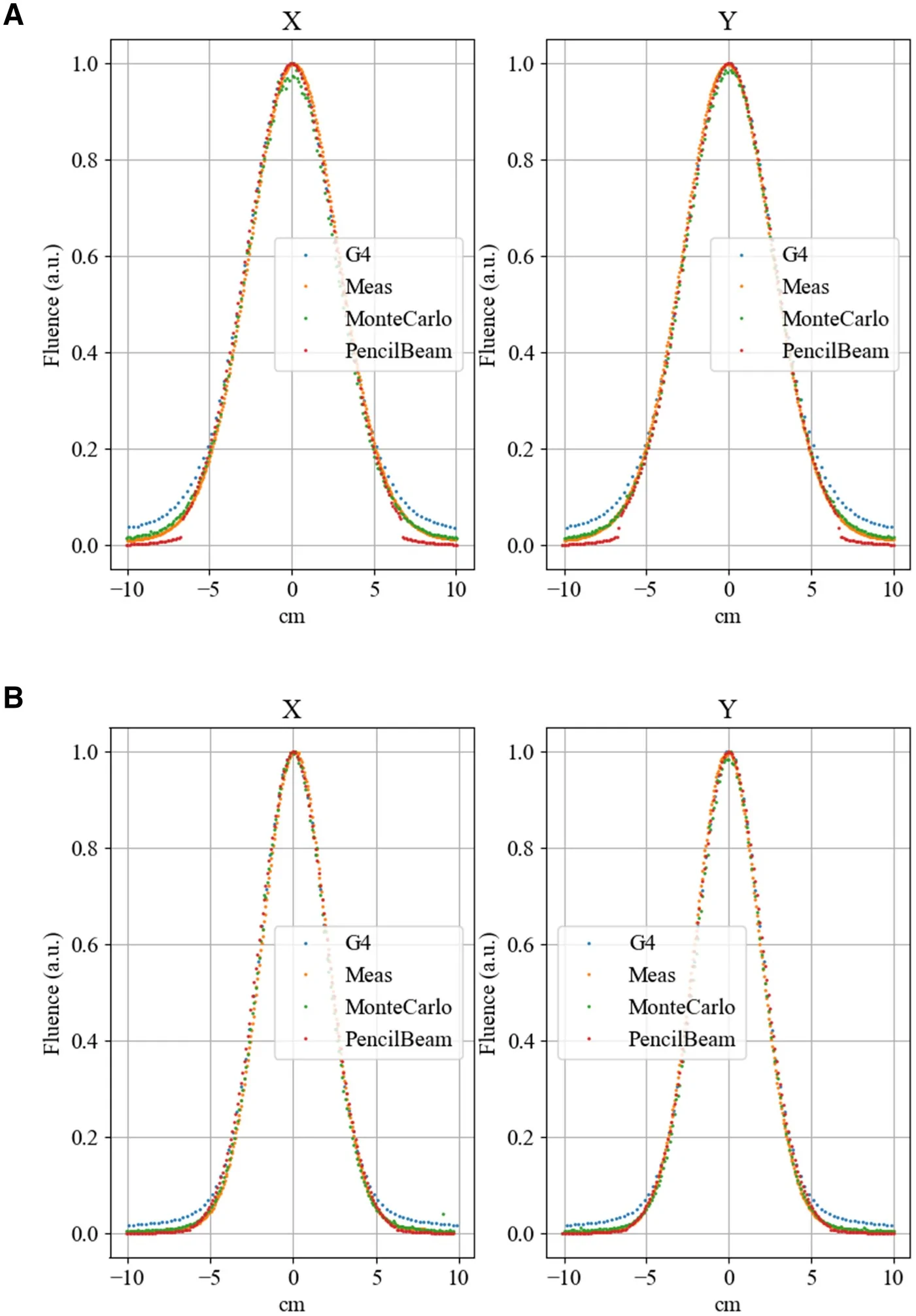
The spot profiles for proton beam of (A) 44.61 MeV and (B) 96.64 MeV. G4 represents the GEANT4 simulation results compared to “Meas,” “PencilBeam,” and “MonteCarlo” which represent measured (“Meas”) and computed (“PencilBeam,” “MonteCarlo”) results with no scintillator. Data for “Meas”, “MonteCarlo,” and “PencilBeam” are provided by UofU HCI Proton Beam Facility.

**Figure 9:**
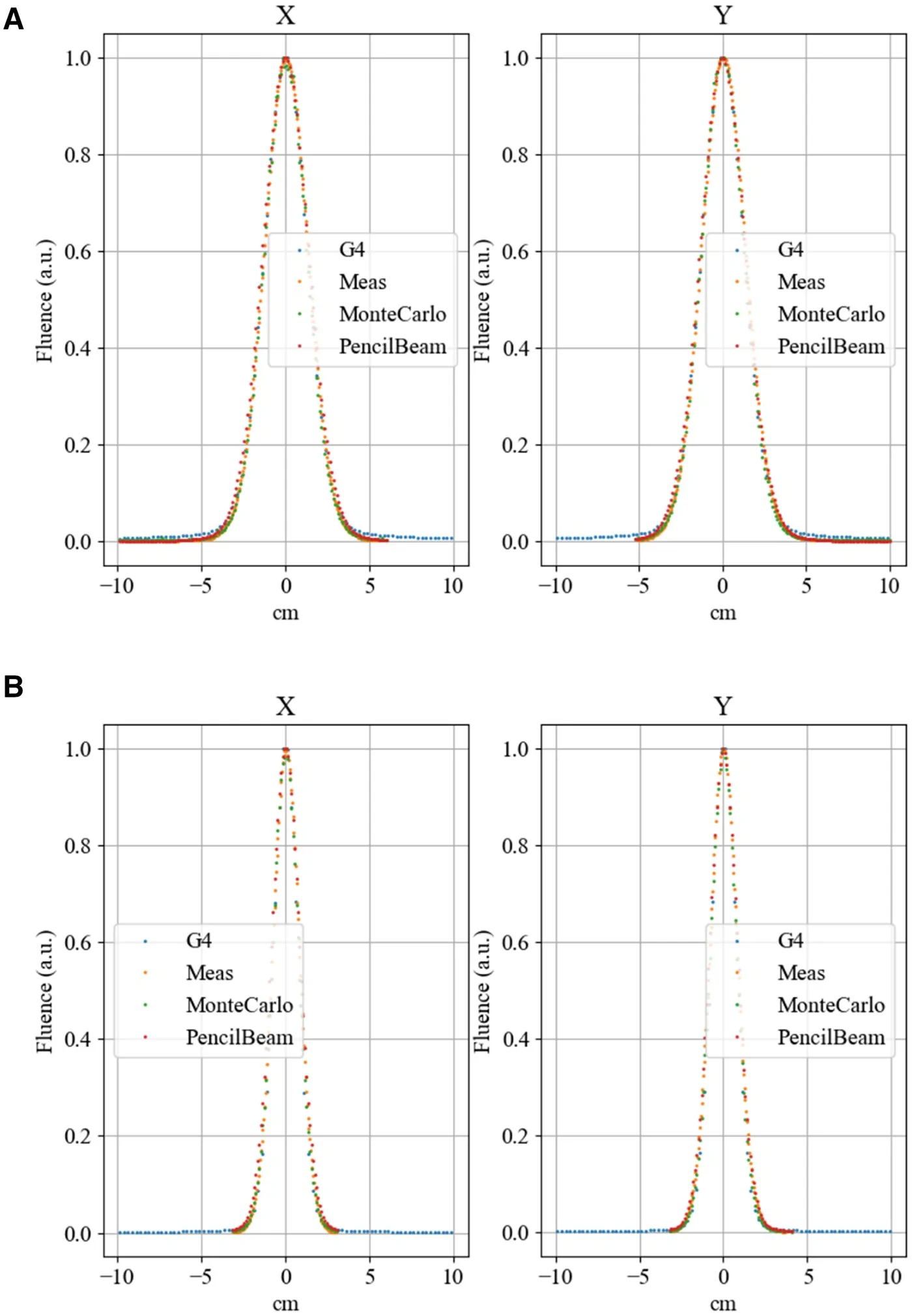
The spot profiles for proton beam of (A) 152.87 Mev and (B) 198.26 MeV. G4 represents the GEANT4 simulation results compared to “Meas”, “PencilBeam,” and “MonteCarlo” which represent measured (“Meas”) and computed (“PencilBeam,” “MonteCarlo”) results with no scintillator. Data for “Meas”, “MonteCarlo,” and “PencilBeam” are provided by UofU HCI Proton Beam Facility.

### X-ray studies and dose calculations

The Instadose+ instrument was used and indicated a near-linear dose increase with increasing electron beam current for our X-ray machine. This is shown in Table 1 with three 10-second X-ray exposures at 4 different current (electron) levels, which were used as a preliminary calibration for subsequent fiber irradiations.

**Table 1:** Dose (mrem) reported by Instadose+ with 12 W X-ray machine exposures.

**Current (** μ **A)**	Trial 1	Trial 2	Trial 3
100	152	164	175
130	244	216	208
170	281	274	286
200	309	308	398

The charge integration values were measured with the scintillating fibers under identical geometry and beam characteristics as the dosimeter. Using the dose values found in Table 1, the total integrated dose (plotted as a function of the charge integration values) shows a translation from the charge integration (in detector units of LSB) to total mrem dose, as in Figure 10.

**Figure 10:**
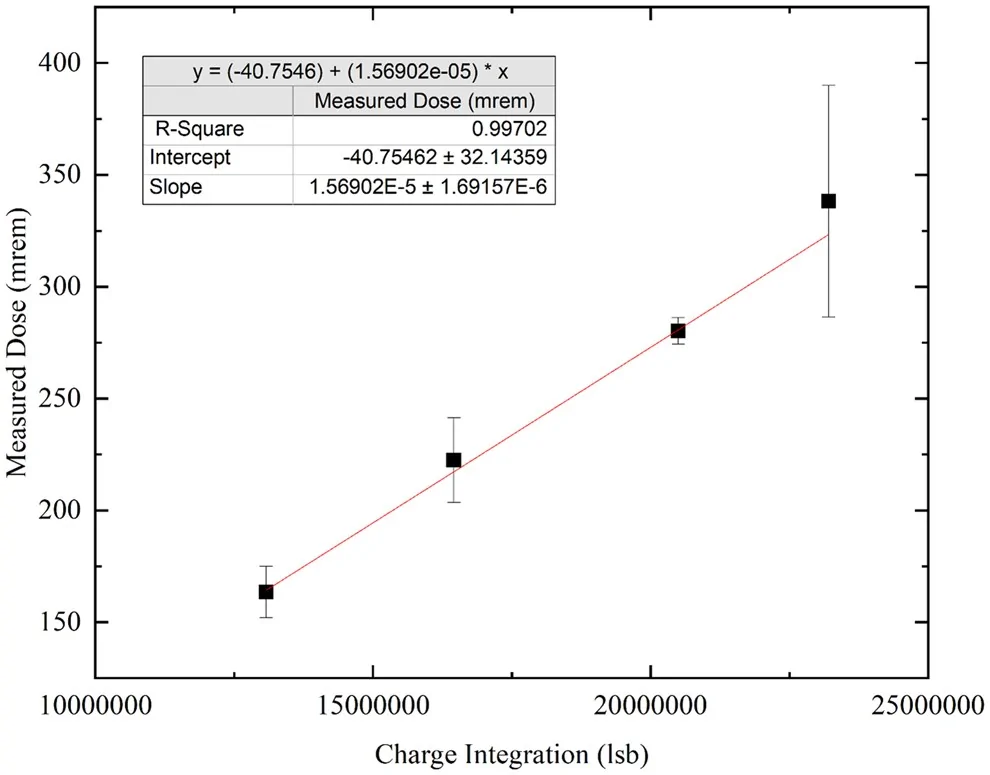
Measured dose from Instadose+ vs measured charge integration by the scintillating fiber for various X-ray currents. The fitted equation represents the expected dose in mrem (along the *y*-axis) as a function of the measured charge integration in LSB (along the *x*-axis).

To quantify the safety interlock, an arbitrary threshold was set at $2\times 10^{7}$ LSB. From Figure 10, this correlates to dose of 273.05 mrem. Total integrated dose is given in Table 2, as well as the deviation from prescribed dose, given as a relative error. The error of prescribed dose increased with increasing current. This was expected as the X-ray machine has a ramp-down period from or to full power. Oscilloscope measurements estimate the X-ray machine ramp-down time to be on the order of 50 ms.

**Table 2:** Overdose delivery compared to prescribed dose from our X-ray machine.

**Current (** μ **A)**	Total (mrem)	Error (%)
100	273.62	0.21
130	273.75	0.26
170	273.90	0.31
200	273.98	0.34

### Proton beam experiments

Our FPGA charge integrations are given in Table 3 for the set of HCI Proton Beam Facility irradiations. Saturation of the readout is present. Thus, there was no expectation of dosimetry during this experimentation at the proton facility. Regardless, we report the average for each energy with the uncertainty representing the standard deviation of all deliveries. Saturation is not desirable; however, it does suggest that the instrumentation is beyond sufficient for detection of radiation. We anticipate that the saturation is attributed to the SiPM (using ∼3000 microcells/channel). Saturation may be eliminated using a different SiPM with more microcells or the use of light attenuators, which can easily be implemented.

**Table 3:** Charge integration for 10 and 100 Monitor Unit (MU) dose prescription.

	10 MU	100 MU
Energy (MeV)	Charge Int (lsb)	Charge Int (lsb)
50	797.6 ± 10.7	6731.3 ± 59.1
100	676.0 ± 15.6	6503.6 ± 72.5
150	723.0 ± 3.0	6916.3 ± 29.2
200	767.3 ± 5.5	7314.3 ± 17.9

Another monitored item was the number of pulses delivered during the experiment (eg, number of pulses recorded by the dosimetry system is recorded in Table 4 for each energy, prescribed dose, and trial). Generally, our detector system recorded more pulses than expected from the treatment system. However, this has been attributed to the final pulse being delivered as a packet of “pulselets,” where the total desired charge is split between 3 and 4 smaller pulses. Regardless, our detector system accurately measured all pulses and pulselets during experimentation.

**Table 4:** Measured vs prescribed pulses for HCI proton beam machine.

	10 MU				100 MU			
Energy (MeV)	Trial			Expected	Trial			Expected
	1	2	3		1	2	3	
50	22	22	23	20	198	198	199	196
100	22	22	22	20	198	198	198	196
150	22	22	22	20	199	198	199	196
200	22	22	22	20	198	198	199	196

## Advances in knowledge

In this work, we advance the understanding and knowledge of a radiation detection system for fast dosimetry. This includes simulation efforts, construction of a prototype detector and readout system, and preliminary testing using the University of Utah Health proton radiotherapy machine in traditional mode. From this effort, we advanced engineering progress toward an instrument that can perform real-time dosimetry of FLASH radiotherapy and provide a safety signal to the radiotherapy machine.

As a result of our work, we have provided a new method of dosimetry for FLASH radiotherapy (ie, more appropriate than the past use of ion chambers). Here, we learn about the use of fiber optics, their use in such a system, and the coupling of optics to electronic readout hardware, the programming of such FPGA hardware, fast timing of signal processing, the incorporation of a safety signal out, as well as the available hardware and software that can be utilized to further this effort. We also advance and report on the understanding of what a dosimetry system would look like using our detection method and potential limitations.

Future work includes addressing signal saturation. This will be achieved through a higher-resolution SiPM or signal light attenuation. Our detector system has shown to be responsive to single pulses. We plan to expand the detector system capability to read and store all measured pulses via an FPGA system, allowing for pulse-by-pulse measurements of dose. Finally, our study only utilized 1 fiber for proof-of-principle on all signal chain and dose computation parts. In the future, a multi-fiber system will be used and tested. Additionally, non-scintillating fibers will also be used to account for Cherenkov radiation. The technology reported in this paper was designed for FLASH RT of protons. However, other applications exist, including conventional RT, radiation beam lines, reactors, and particle accelerators.

## Conclusions

In our work, we simulate and test a prototype instrument for FLASH RT dosimetry. Our results are promising but also point to areas for improvement. We utilized GEANT4 simulations that advised that a 1-mm scintillator has very little significance in altering the primary beam characteristics for a therapeutic proton beam in FLASH RT mode. Strong linearity of dose appeared throughout testing on our instrument. It is expected that the interlock system proposed here will be a requirement for safety functionality in the FLASH regime testing.

Our irradiation experiments at HCI led us to believe that signal saturation had occurred with the detector system. Regardless, our instrument adequately performed measurements to that point, including tracking delivered pulses during the experiment. Our work will require a dosimetry comparison against known and verified dosimetry materials and methods. The dosimetry system proposed here allows for pulse-by-pulse monitoring of the exit beam. Lessons learned during this experimental campaign are to be implemented into the dosimetry system engineering and testing methodology.

## Supplementary Material

tqag152_Supplementary_Data

## References

[1] Schüler E , Trovati S, King G, et al Experimental platform for ultra-high dose rate FLASH irradiation of small animals using a clinical linear accelerator. Int J Radiat Oncol Biol Phys. 2017;97:195-203. 10.1016/j.ijrobp.2016.09.018 27816362

[2] Diffenderfer ES , Verginadis II, Kim MM, et al Design, implementation, and in vivo validation of a novel proton FLASH radiation therapy system. Int J Radiat Oncol Biol Phys. 2020;106:440-448. 10.1016/j.ijrobp.2019.10.049 PMC7325740 31928642

[3] Lempart M , Blad B, Adrian G, et al Modifying a clinical linear accelerator for delivery of ultra-high dose rate irradiation. Radiother Oncol. 2019;139:40-45. 10.1016/j.radonc.2019.01.031 30755324

[4] Bazalova-Carter M , Esplen N. On the capabilities of conventional x-ray tubes to deliver ultra-high (FLASH) dose rates. Med Phys. 2019;46:5690-5695. 10.1002/mp.13858 31600830

[5] Esplen N , Mendonca MS, Bazalova-Carter M. Physics and biology of ultrahigh dose-rate (FLASH) radiotherapy: a topical review. Phys Med Biol. 2020;65:23TR03. 10.1088/1361-6560/abaa28 32721941

[6] DE Marzi L , et al Exploiting the full potential of proton therapy: an update on the specifics and innovations towards spatial or temporal optimisation of dose delivery. Cancer/Radiotherapie. 2020;24:6-7. 10.1016/j.canrad.2020.06.003 32753235

[7] Chow JCL , Ruda HE. Flash radiotherapy: innovative cancer treatment. Encyclopaedia. 2023;3 3:808-823. 10.3390/encyclopedia3030058

[8] Harrington KJ. Ultrahigh dose-rate radiotherapy: next steps for flash-RT. Clin Cancer Res. 2019;25:3-5. 10.1158/1078-0432.CCR-18-1796 30093447

[9] Gianfaldoni S , Gianfaldoni R, Wollina U, et al An overview on radiotherapy: from its history to its current applications in dermatology. Open Access Maced J Med Sci. 2017;5:521-525. 10.3889/oamjms.2017.122 PMC5535674 28785349

[10] Hughes JR , Parsons JL. Flash radiotherapy: current knowledge and future insights using proton-beam therapy. Int J Mol Sci. 2020;21:1. 10.3390/ijms21186492 PMC7556020 32899466

[11] Nesteruk KP , Psoroulas S. Flash irradiation with proton beams: beam characteristics and their implications for beam diagnostics. Applied Sciences (Switzerland). 2021;11:2170. 10.3390/app11052170

[12] Folkerts MM , Abel E, Busold S, et al A framework for defining FLASH dose rate for pencil beam scanning. Med Phys. 2020;47:6396-6404. 10.1002/mp.14456 PMC7894358 32910460

[13] Bourhis J , Sozzi WJ, Jorge PG, et al Treatment of a first patient with FLASH-radiotherapy. Radiother Oncol. 2019;139:18-22. 10.1016/j.radonc.2019.06.019 31303340

[14] Favaudon V , et al Ultrahigh dose-rate FLASH irradiation increases the differential response between normal and tumour tissue in mice. Radiat Toxicity. 2014;6:245. 10.1126/scitranslmed.3008973 25031268

[15] Mascia AE , Daugherty EC, Zhang Y, et al Proton FLASH radiotherapy for the treatment of symptomatic bone metastases: the FAST-01 nonrandomized trial. JAMA Oncol. 2023;9:62-69. 10.1001/jamaoncol.2022.5843 PMC9589460 36273324

[16] Beyreuther E , Brand M, Hans S, et al Feasibility of proton FLASH effect tested by zebrafish embryo irradiation. Radiother Oncol. 2019;139:46-50. 10.1016/j.radonc.2019.06.024 31266652

[17] Mazal A , Prezado Y, Ares C, et al FLASH and minibeams in radiation therapy: the effect of microstructures on time and space and their potential application to protontherapy. Br J Radiol. 2020;93:20190807. 10.1259/bjr.20190807 PMC7066940 32003574

[18] Ashraf MR , Rahman M, Zhang R, et al Dosimetry for FLASH radiotherapy: a review of tools and the role of radioluminescence and cherenkov emission. Beams 2020;106:3

[19] Romano F , Subiel A, McManus M, et al Challenges in dosimetry of particle beams with ultra-high pulse dose rates. J Phys: Conf Ser. 2020;1662:012028. 10.1088/1742-6596/1662/1/012028

[20] Jolly S , Owen H, Schippers M, et al Technical challenges for FLASH proton therapy. Phys Med. 2020;78:71-82. 10.1016/j.ejmp.2020.08.005 32947086

[21] Di Martino F , Barca P, Barone S, et al FLASH radiotherapy with electrons: Issues related to the production, monitoring, and dosimetric characterization of the beam. Front Phys. 2020;8:1. 10.3389/fphy.2020.570697

[22] McManus M , Romano F, Lee ND, et al The challenge of ionisation chamber dosimetry in ultra-short pulsed high dose-rate very high energy electron beams. Sci Rep. 2020;10:9089. 10.1038/s41598-020-65819-y PMC7270129 32493952

[23] Petersson K , Jaccard M, Germond J-F, et al High dose-per-pulse electron beam dosimetry-a model to correct for the ion recombination in the advanced Markus ionization chamber. Med Phys. 2017;44:1157-1167. 10.1002/mp.12111 28094853

[24] Harrison N , Charyyev S, Oancea C, et al Characterizing devices for validation of dose, dose rate, and LET in ultra high dose rate proton irradiations. Med Phys. 2024;51:8411-8422. 10.1002/mp.17359 39153223

[25] Favaudon V , Lentz J-M, Heinrich S, et al Time-resolved dosimetry of pulsed electron beams in very high dose-rate, FLASH irradiation for radiotherapy preclinical studies. Nucl Instrum Methods Phys Res A. 2019;944:162537. 10.1016/j.nima.2019.162537

[26] Bourgouin A , Schüller A, Hackel T, et al Calorimeter for real-time dosimetry of pulsed ultra-high dose rate electron beams. Front Phys. 2020;8:1. 10.3389/fphy.2020.567340

[27] Winterhalter C , Togno M, Nesteruk KP, et al Faraday cup for commissioning and quality assurance for proton pencil beam scanning beams at conventional and ultra-high dose rates. Phys Med Biol. 2021;66:1-10. 10.1088/1361-6560/abfbf2 33906166

[28] Ashraf MR , Rahman M, Zhang R, et al Technical note: single-pulse beam characterization for FLASH-RT using optical imaging in a water tank. Med Phys. 2021;48:2673-2681. 10.1002/mp.14843 PMC10771323 33730367

[29] Jørgensen EB , Johansen JG, Overgaard J, et al A high-Z inorganic scintillator-based detector for time-resolved in vivo dosimetry during brachytherapy. Med Phys. 2021;48:7382. 10.1002/mp.15257 34586641

[30] Zou W , Diffenderfer ES, Ota K, et al Characterization of a high-resolution 2D transmission ion chamber for independent validation of proton pencil beam scanning of conventional and FLASH dose delivery. Med Phys. 2021;48:3948-3957. 10.1002/mp.14882 33843065

[31] Ashraf MR , Rahman M, Cao X, et al Individual pulse monitoring and dose control system for pre-clinical implementation of FLASH-RT. Phys Med Biol. 2022;67:1-27. 10.1088/1361-6560/ac5f6f PMC10305796 35313290

[32] Christensen JB , Togno M, Nesteruk KP, et al Al_2_O_3_: C optically stimulated luminescence dosimeters (OSLDs) for ultra-high dose rate proton dosimetry. Phys Med Biol. 2021;66:1-11. 10.1088/1361-6560/abe554 33571973

[33] Rahman M , Ashraf MR, Zhang R, et al Spatial and temporal dosimetry of individual electron FLASH beam pulses using radioluminescence imaging. Phys Med Biol. 2021;66:1-11. 10.1088/1361-6560/ac0390 PMC10468779 34015774

[34] Chen L , O’Keeffe S, Chen S, et al Investigation of YAG: Ce-Based optical fibre sensor for use in Ultra-Fast external beam radiotherapy dosimetry. J Lightwave Technol. 2019;37:4741-4747. 10.1109/JLT.2019.2919605

[35] Wang Z , et al Thin scintillators for ultrafast hard X-ray imaging. Photon Counting Applications 2015, 9504 May 2015, 95040N. 2015. 10.1117/12.2178420

[36] Alsanea F , Therriault-Proulx F, Sawakuchi G, et al A real-time method to simultaneously measure linear energy transfer and dose for proton therapy using organic scintillators. Med Phys. 2018;45:1782-1789. 10.1002/mp.12815 PMC9624230 29446078

[37] Pogue BW , Krishnaswamy V, Jermyn M, et al Real time radiotherapy verification with Cherenkov imaging: development of a system for Beamlet verification. J Phys: Conf Ser. 2017;847:012042. 10.1088/1742-6596/847/1/012042

[38] Beddar S , Archambault L, Sahoo N, et al Exploration of the potential of liquid scintillators for real-time 3D dosimetry of intensity modulated proton beams. Med Phys. 2009;36:1736-1743. 10.1118/1.3117583 PMC2832031 19544791

[39] Ferrero V , Fiorina E, Morrocchi M, et al Online proton therapy monitoring: clinical test of a silicon-photodetector-based in-beam PET. Sci Rep. 2018;8:4100. 10.1038/s41598-018-22325-6 PMC5840345 29511282

[40] Siddique S , Ruda HE, Chow JCL. FLASH radiotherapy and the use of radiation dosimeters.Cancers (Basel). 2023;15:1-16. 10.3390/cancers15153883 PMC10417829 37568699

[41] Vanreusel V , Gasparini A, Galante F, et al Point scintillator dosimetry in ultra-high dose rate electron ‘FLASH’ radiation therapy: a first characterization. Phys Med. 2022;103:127-137. 10.1016/j.ejmp.2022.10.005 36302279

[42] Vanreusel V , Gasparini A, Galante F, et al Optically stimulated luminescence system as an alternative for radiochromic film for 2D reference dosimetry in UHDR electron beams. Phys Med. 2023;114:103147. 10.1016/j.ejmp.2023.103147 37804712

[43] Morrocchi M , Pensavalle JH, Ciarrocchi E, et al Experimental characterization and monte carlo simulation of scintillator detectors in online electron FLASH radiotherapy dosimetry. J Inst. 2022;17:P09005. 10.1088/1748-0221/17/09/P09005

[44] Moxtek^®^ Tub-Data-1013, Rev I Subject to technical change without notice MAGPRO^TM^.

[45] Shen J , Ding X, Charyyev S, et al Time structures of proton pencil beam scanning delivery on a microsecond scale measured with a pixelated semiconductor detector Timepix3. J Appl Clin Med Phys. 2024;25:e14486. 10.1002/acm2.14486 PMC11492390 39137008

[46] Vilches-Freixas G, Unipan M, Rinaldi I, et al Beam commissioning of the first compact proton therapy system with spot scanning and dynamic field collimation. Br. J. Radiol. 2020;93:20190598. 10.1259/bjr.20190598 PMC7066978 31782941

[47] Mevion, S250i Hyperscan. Accessed October 18, 2023. https://www.mevion.com/products/mevion-s250i-hyperscan-adaptive-aperturetm

[48] Mevion. HCI Physics Course in Internal. Mevion, 2020.

[49] Agostinelli S , Allison J, Amako K, et al GEANT4 - a simulation toolkit. Nucl Instrum Methods Phys Res A. 2003;506:3-250. 10.1016/S0168-9002(03)01368-8

[50] Luxium Solutions, BCF-12 Scintillating Fiber. Accessed October 4, 2023. https://www.luxiumsolutions.com/sites/default/files/2021-11/Fiber-Product-Sheet.pdf

[51] Industrial Fiber Optics. Accessed October 4, 2023. https://i-fiberoptics.com/

[52] Hamamatsu, MPPC Module. Accessed September 24, 2023. https://www.hamamatsu.com/us/en/product/optical-sensors/mppc/mppc-module/C13369-3050EA-04.html

[53] Caen. DT5550 32 Channel Programmable Readout System. Accessed September 24, 2023. https://www.caen.it/products/dt5550/

