# Supplementary material for: Development of in situ dosimetry for FLASH proton radiotherapy via organic scintillating fibers
Source: Br J Radiol. 2026 Jun 19;99(1186):2117–28. doi: 10.1093/bjr/tqag152 (PMC13623240; doi:10.1093/bjr/tqag152)
Supplement: Supplementary file 1 [file tqag152_supplementary_data.zip › supplementary material .docx]

Development of in situ dosimetry for FLASH proton radiotherapy via organic scintillating fibers

**Supplementary Material**

**Figure SM1:** 12 W desktop X-ray machine from Moxtek INC. Beam exit is located on the left, surrounded by copper.

**Figure SM2:** The treatment vault and gantry of the Mevion S250i Synchrocyclotron at Huntsman Cancer Institute.

**Figure SM3:** Schematic of the logic level shifter for communication between the FPGA and X-ray machine.
